# How to select elderly colorectal cancer patients for surgery: a pilot study in an Italian academic medical center

**DOI:** 10.7497/j.issn.2095-3941.2015.0084

**Published:** 2015-12

**Authors:** Giampaolo Ugolini, Francesco Pasini, Federico Ghignone, Davide Zattoni, Maria Letizia Bacchi Reggiani, Daniele Parlanti, Isacco Montroni

**Affiliations:** Department of Medical and Surgical Sciences, University of Bologna, S. Orsola Malpighi Hospital, Bologna 40138, Italy

**Keywords:** Elderly, geriatric assessment, surgical oncology, risk assessment, screening tools, colorectal cancer (CRC)

## Abstract

**Objective:**

Cancer is one of the most common diagnoses in elderly patients. Of all types of abdominal cancer, colorectal cancer (CRC) is undoubtedly the most frequent. Median age at diagnosis is approximately 70 years old worldwide. Due to the multiple comorbidities affecting elderly people, frailty evaluation is very important in order to avoid over- or under-treatment. This pilot study was designed to investigate the variables capable of predicting the long-term risk of mortality and living situation after surgery for CRC.

**Methods:**

Patients with 70 years old and older undergoing elective surgery for CRC were prospectively enrolled in the study. The patients were preoperatively screened using 11 internationally-validated-frailty-assessment tests. The endpoints of the study were long-term mortality and living situation. The data were analyzed using univariate Cox proportional-hazard regression analysis to verify the predictive value of score indices in order to identify possible risk factors.

**Results:**

Forty-six patients were studied. The median follow-up time after surgery was 4.6 years (range, 2.9-5.7 years) and no patients were lost to follow-up. The overall mortality rate was 39%. Four of the patients who survived (4/28, 14%) lost their functional autonomy. The preoperative impaired Timed Up and Go (TUG), Eastern Cooperative Group Performance Status (ECOG PS), Instrumental Activities of Daily Living (IADLs), Vulnerable Elders Survey (VES-13) scoring systems were significantly associated with increased long term mortality risk.

**Conclusion:**

Simplified frailty-assessing tools should be routinely used in elderly cancer patients before treatment in order to stratify patient risk. The TUG, ECOG-PS, IADLs and VES-13 scoring systems are potentially able to predict long-term mortality and disability. Additional studies will be needed to confirm the preliminary data in order to improve management strategies for oncogeriatric surgical patients.

## Introduction

The number of cancer cases is steadily increasing, and malignancies are expected to become the first cause of death worldwide in the upcoming decades^1^. The number of elderly people is rapidly increasing worldwide and 50% of all cancer cases and 70% of cancer related deaths occur in this group^2^. This is particularly true for intra-abdominal malignancies which are often diagnosed in elderly patients^3^. Colorectal cancer (CRC) is the third most common neoplasm worldwide with 1.36 million new cases/year^4^, and half of the new diagnoses are made in people 70 years old or older. Multimodal treatment and multidisciplinary teams have been widely recognized as a fundamental approach in treating cancer patients^5^^,^^6^; however, despite progress in medical and radiation oncology, surgery remains the cornerstone of treatment and the only chance for cure of many CRCs.

A variety of challenges need to be faced when taking care of elderly patients, mainly related to the complexity of this particular group of patients^7^. Age ‘*per se*’ should not be considered a contraindication for accessing treatment since several studies worldwide have failed to prove its direct correlation with any risk of postoperative complications^8^. Elderly individuals should not be denied radical surgery due to their chronological age as studies showed that elderly people have similar cancer-free survival to their younger counterparts^9^^,^^10^.

Many factors, such as comorbidities, sarcopenia, polypharmacy and nutritional status^11^, instead, play a significant role in an elderly patient’s life expectancy and are able to predict whether patients will tolerate cancer treatment. Geriatric evaluation should be carried out before treatment decisions are made in order to assess whether the patient’s remaining life expectancy will be reduced by cancer or by the patient’s coexisting conditions. In particular, preservation of functional independence is considered one of the most important patient-reported end-point (frequently over the disease free survival) and for this reason it becomes crucial to investigate predictors for long-term status maintenance.

Comprehensive Geriatric Assessment (CGA) is recognized as the best tool for preoperatively evaluating oncogeriatric patients (OP)^12^^,^^13^ but, unfortunately, it is time consuming and hence difficult to use in a busy surgical clinical practice. Recent studies have sought simplified tools of rapid execution^14^^,^^15^ capable of assessing the surgical risk in elderly patients undergoing surgery for cancer. The preoperative assessment in elderly cancer patients study (PACE) showed that Instrumental Activities of Daily Living (IADLs) and Performance Status (PS) were associated with a 50% increase in the relative risk of postoperative complications and extended hospital stay^16^. The preoperative risk estimation for oncogeriatric patients (PRE-OP) study has shown that Timed Up and Go (TUG), the American Society of Anesthesiologists (ASA) score and Nutritional Risk Screening (NRS) can predict short-term postoperative complications and mortality^17^.

The aim of the current pilot study was to investigate whether some rapid execution screening tools for functional status were also able to predict long-term mortality in elderly patients undergoing surgery for CRC.

## Materials and methods

### Patient selection

Patients with 70 years old or older who underwent surgery for CRC were prospectively enrolled in the study. Patients requiring emergent surgery and patients who were unable to give written informed consent were excluded. Patients were recruited from the General Surgery Department of S. Orsola-Malpighi Hospital (Bologna, Italy) between September 2009 and June 2012. The study protocol was reviewed and approved by the institutional ethics committee.

### Screening tools

Within 10 days prior to their surgery, the patients enrolled were evaluated in order to assess their preoperative functional status using a cluster of screening tools.

TUG^14^, Activity of Daily Living (ADL)^18^, IADLs^19^ and the Eastern Cooperative Oncology Group Performance Status (ECOG PS)^20^ were carried out to evaluate functional status. The Mini Mental State Examination (MMSE)^21^ was used to assess cognitive function. The Groningen Frailty Index (GFI)^22^ and the Vulnerable Elders Survey (VES-13)^23^ were used to identify possible vulnerable and frail individuals. Fatigue was analysed using Brief Fatigue Inventory (BFI)^24^, and depression with the Geriatric Depression Scale (GDS)^25^. Nutritional status was assessed using NRS^26^, which identifies mildly, moderately and severely impaired nutritional statuses. The ASA score determined by an anesthesiologist was also reported. The screening tools are summarized in **Table 1**. Data about living situation were also collected, considering it as a surrogate index of long term functional status after surgical procedure.

**Table 1:** Screening tools used in the study and frequencies of impaired results

Test	Score range	Cut-off*	Impaired** (%)
Timed Up & Go (TUG)	NA	>20 s	11 (23.9)
Activities of Daily Living (ADL)	0-6	<6	11 (23.9)
Instrumental Activities of Daily Living (IADLs)	0-8	<8	18 (39.1)
ECOG Performance Status (ECOG-PS)	0-4	>1	10 (21.7)
Mini Mental State Examination (MMSE)	0-30	<25	23 (50.0)
Groeningen Frailty Index (GFI)	0-15	≥4	24 (52.2)
Vulnerable Elderly Survey - 13 (VES-13)	0-10	≥3	20 (43.5)
Brief Fatigue Inventory (BFI)	0-10	>3	22 (47.8)
Geriatric Depression Scale (GDS)	0-15	>5	12 (26.1)
Nutritional Risk Screening (NRS)	NA	NA	16 (34.8)
American Society of Anesthesiologists score (ASA)	1-5	≥3	32 (69.6)

*, Cut-off for the impaired test; **, No. of impaired results for each test. NA, not available.

### Data collection

After early postoperative follow-up in the outpatient clinic, long term follow up was assessed by contacting previously operated on patients or their caregivers in order to retrieve information regarding mortality and current living situation, asking whether patients were living independently, with family members/care-givers or in a nursing home.

### Endpoints

The primary endpoint was to evaluate the capacity of validated preoperative screening tools to predict long term mortality.

The secondary endpoint was evaluation of long term living situation.

### Statistical analysis

The categorical data were expressed as numbers (percentages), and the continuous variables as means and standard deviations. The results of the preoperative tests were divided into “normal” and “impaired”, according to the cut-offs in the literature (**Table 1**). Estimated long-term survival in the different scores was assessed by the Kaplan-Meier method. Univariate Cox proportional hazard regression analysis was conducted to verify the predictive value of score index, age and sex. A *P* value of <0.05 (2-sided) was considered significant. A multivariate analysis was generated but was not reported since it was not significant because of the small number of patients enrolled in the study. Statistical analysis was carried out using Stata Statistical Software release 14 (College Station, TX, StataCorp LP).

## Results

A total of 46 CRC patients were enrolled in this study, 24 males and 22 females. The median age of our patients was 80.52±6.68 years. Before surgery, the majority of patients were living independently (*n*=35; 76%) while 11 (24%) were institutionalized or living with assistance from family members or care-givers, because of cognitive/functional status impairment.

For each of the performed screening tool we obtained the following frequencies of impaired results (cut offs are shown in **Table 1**): VES-13: 20; MMSE: 23; ADL: 11; IADLs: 18; GDS: 12; ECOG PS: 10; GFI: 24; BFI: 32; NRS: 16; TUG: 11.

The median follow-up time after surgery was 4.6 years (range, 2.9-5.7 years) and no patients were lost to follow-up.

**Figure 1:**
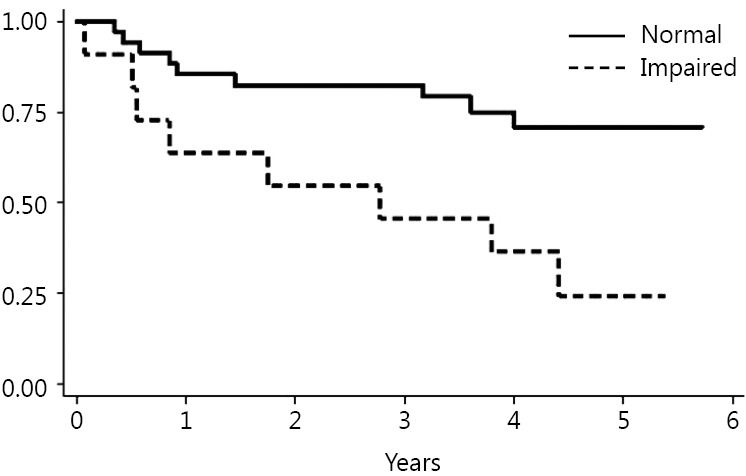
Timed Up & Go (TUG).

**Figure 2:**
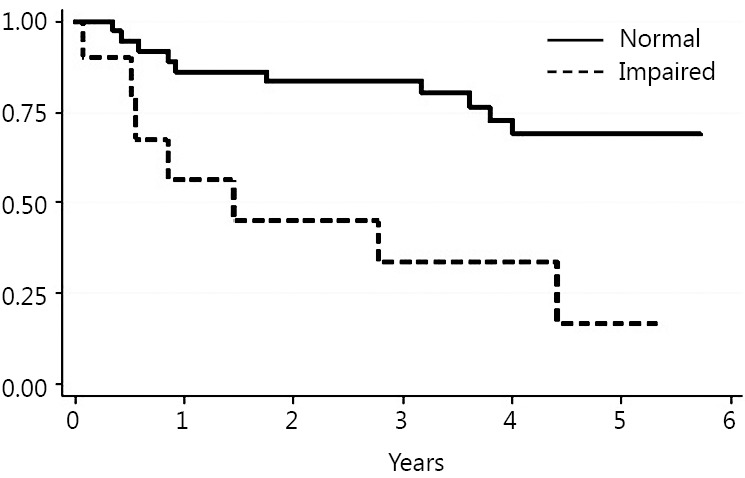
ECOG Performance Status (ECOG-PS).

**Figure 3:**
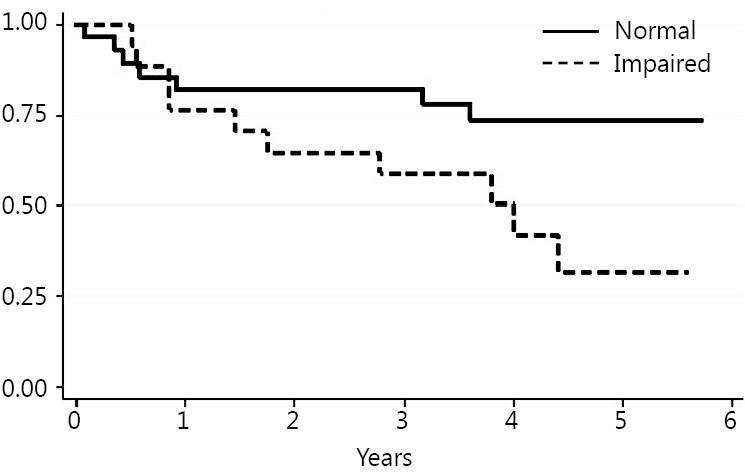
Instrumental Activities of Daily Living (IADLs).

**Figure 4:**
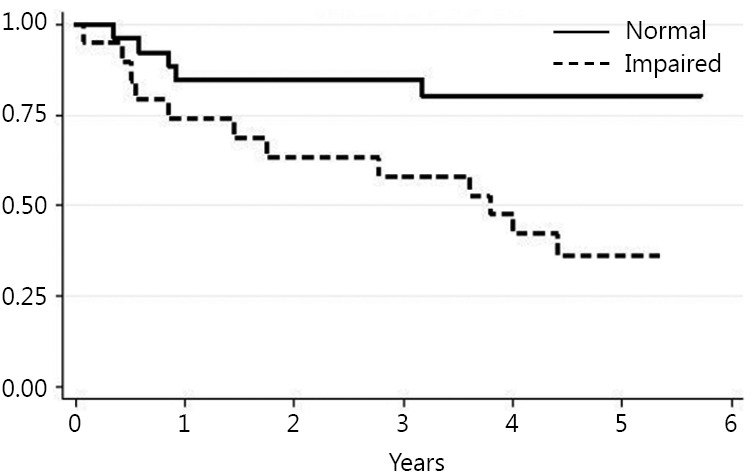
Vulnerable Elderly Survey - 13 (VES-13).

Death occurred in 18 patients (39%) during follow up. The Kaplan Meier method was used to evaluate the ability of each test to predict mortality. The TUG, ECOG-PS, IADLs and NRS results statistically correlated with long-term mortality risk (**Figures 1****,****2****,****3****,****4**). Univariate Cox proportional hazard regression was undertaken to verify the predictive value of the proposed scores; the results are reported in **Table 2**. Age was associated with an increased risk per year (HR =1.10; *P*=0.003; 95% CI, 1.03-1.17). Gender was not associated with an increased mortality rate. There were no significant differences between genders.

**Table 2:** Univariate Cox regression

Test	HR	*P*	95% CI
Vulnerable Elderly Survey - 13 (VES-13)	3.664	0.015*	1.288-10.416*
Mini Mental State Examination (MMSE)	2.680	0.064	0.943-7.615
Activities of Daily Living (ADL)	1.713	0.313	0.601-4.880
Instrumental Activities of Daily Living (IADLs)	2.716	0.044*	1.028-7.177*
Geriatric Depression Scale (GDS)	1.710	0.291	0.631-4.634
ECOG Performance Status (ECOG-PS)	4.139	0.004*	1.564-10.951*
Groeningen Frailty Index (GFI)	2.265	0.108	0.836-6.136
Brief Fatigue Inventory (BFI)	2.138	0.124	0.811-5.638
American Society of Anesthesiologists score (ASA)	2.347	0.180	0.674-8.171
Nutritional Risk Screening (NRS)	2.264	0.094	0.871-5.884
Timed Up & Go (TUG)	3.507	0.010*	1.350-9.113*

*, Significant results.

At univariate analysis, four screening tools were able to predict an increased risk of death in this group of patients: VES-13 (HR =3,664; *P*=0.015; 95% CI, 1.29-10.42), IADL (HR =2,716; *P*=0.044; 95% CI, 1.03-7.18), ECOG-PS (HR =4,139; *P*=0.004; 95% CI, 1.56-10.95) and TUG (HR =3,507; *P*=0.010; 95% CI, 1.35-9.11). Of the patients who survived at follow-up, only a minority (4/28, 14.2%) had changed their living situations from “independent” to “dependent” while 24 patients who were living independently before their surgery maintained their status.

Due to the small number of surviving patients who postoperatively lost independence during the study period, no correlation with preoperative assessment tools was evaluated statistically, even if the vast majority of long-term living patients conserved their status of independence in daily activities.

## Discussion

Since the number of elderly patients affected by cancer is growing together with life expectancy, geriatric oncology has become an important area of research, and surgical treatment for this subset of patients still represents an open area for debate. It is nowadays accepted that chronological age is no longer a limitation for surgery, and it should no longer be considered as a surrogate for fitness for surgery.

Elderly cancer patients, if appropriately treated, can have an excellent survival rate after curative surgery^9^^,^^10^^,^^27^ as confirmed by this study, showing a notable long-term survival rate reported for octogenarians (61% at 4.6 years). Thus, the key to reaching optimal outcomes is to differentiate between biological and chronological age, tailoring the right treatment for the right patient.

Physical and cognitive status, together with comorbidities and frailty, should be accurately assessed prior to surgery in order to identify patients who can tolerate standard therapies.

Surgeons have historically been reluctant to incorporate time-consuming and complex screening tools in their practice, and CGA has never been widely adopted in daily clinical activity. In this pilot study, the ability of 11 simplified screening tools to predict long-term mortality was evaluated.

In the geriatric setting, TUG, VES-13, ECOG-PS and IADLs have already been widely recognized but they have never been utilized in a surgical routine in order to predict long-term results^15^^,^^23^^,^^28^^,^^29^. Being able to stratify patients could improve outcomes, in particular for the most vulnerable patients who could benefit from pre-habilitation^30^^,^^31^ or a tailored surgical strategy, considering their quality of life (QOL) as the most important endpoint. Our study highlighted that even in the surgical setting these screening tools could be useful to identify patients at higher risk of mortality. Including TUG, VES-13, ECOG-PS and IADLs in the routine practice could enhance dedicated pathways for oncogeriatric patients, putting surgical oncologists in better condition to distinguish those who could really benefit from a surgical treatment.

EUROCARE-5 study has recently provided interesting data in terms of long term survival for patients affected by colon cancer. Patients aged 65-74 years old had a 59.5% rate of cancer-related survival, while youngers (45-54 years old) had a 62.4% rate^32^. This trend in survival was confirmed by the rate in our cohort study where it was found to be 61%.

Significant data were provided by a recent study by Booth and colleagues that showed that elderly patients who underwent liver resection for colorectal metastasis have similar long term survival compared with their younger counterparts^33^.

Another recent population-based study promoted in the Netherlands showed that elderly patients who underwent surgery for pancreatic cancer who survived 90 days postoperatively exhibited an overall survival close to younger patients^34^.

To stratify elderly according to frailty status becomes crucial: this is because fit-for-surgery patients will be more prone to have excellent long term survival rate, while those who are at higher risk for complications may be offered alternative therapeutic strategies, avoiding overtreatment. For this reason we strongly believe that preoperative screening tools, such as TUG, VES-13, ECOG-PS and IADLs could have a pivotal role in a comprehensive evaluation of OPs candidate for surgical treatment.

Furthermore, the vast majority of those patients who survived and who were alive at the end of the follow-up, conserved their preoperative living situation. Our study did not identify surgical variables able to predict living situation impairment; for this reason, living situation needs to be deeply explored in future larger longitudinal studies since functional status preservation could represent the most important endpoint from the perspective of the elderly patient^35^.

The limitations of the study include the small number of patients enrolled and a lack of information regarding the long-term QOL in our surgical OPs. In fact, living situation is only a surrogate indicator and does not take into account many factors which contribute to determining QOL. Furthermore, in actual practice, it is not possible to rely solely on tests to determine management strategies without thoroughly considering patient perspective. When evaluating elderly patients, it is crucial to openly discuss the options, pros and cons in the presence of the caregivers in order to establish a ‘therapeutic alliance’. Future studies will be needed to establish and validate the path for obtaining a holistic evaluation of the oncogeriatric population which could be adopted in everyday practice in order to improve patient selection for surgical treatment. These steps are essential for planning personalized treatment in order to maximize benefits and reduce risks, offering better treatment to patients unfit for standard therapy.
